# Salivary Testosterone and Cortisol as Biomarkers for the Diagnosis of Sarcopenia and Sarcopenic Obesity in Community-Dwelling Older Adults

**DOI:** 10.3390/biology10020093

**Published:** 2021-01-27

**Authors:** Angela Diago-Galmés, Carlos Guillamón-Escudero, Jose M. Tenías-Burillo, Jose M. Soriano, Julio Fernández-Garrido

**Affiliations:** 1 Hospital Universitario de La Plana, 12540 Villareal, Spain; angela94dg@gmail.com; 2 Hospital General Universitari de Castelló, 12004 Castellón, Spain; carlos_ge@hotmail.es; 3 Department of Preventive Medicine, Hospital Pare Jofré, 46017 Valencia, Spain; Tenias_jma@gva.es; 4 Food & Health Lab, Institute of Materials Science, University of Valencia, 46980 Valencia, Spain; 5 Joint Research Unit on Endocrinology, Nutrition and Clinical Dietetics, University of Valencia-Health Research Institute La Fe, 46026 Valencia, Spain; 6 Department of Nursing, Faculty of Nursing and Podiatry, University of Valencia, 46001 Valencia, Spain; Julio.Fernandez@uv.es

**Keywords:** testosterone, cortisol, community older adults

## Abstract

**Simple Summary:**

Sarcopenia (S) and sarcopenic obesity (SO) are diseases that increasingly affect society and constitute an important part of the pathologies that affect elderly due to the high life expectancies of the current population. The search for accessible, inexpensive, and noninvasive biomarkers that can help diagnose these diseases quickly and easily is a new field that deserves an in-depth study. The aim of this study was to find relationships between salivary cortisol (C) and testosterone (T) levels and these pathologies’ appearance in 190 community-dwelling men and women over 65 years old. The results reflect a relationship between salivary T and the age of the participants with differences by sex, and a relationship was found between lower levels of T and a greater presence of S and SO. Despite the significant results obtained, more studies are necessary to determine a potential panel of salivary biomarkers for the study of S and SO.

**Abstract:**

Nowadays, the appearance of sarcopenia (S) or sarcopenic obesity (SO) is related to aging. According to the criteria of the European Working Group on Sarcopenia in Older People (EWGSOP), the feasibility of using salivary cortisol and testosterone levels was analyzed as diagnostic biomarkers of S or SO. One hundred and ninety non-institutionalized people aged ≥65 years were studied, independent of the activities of daily living (ADLs) (Barthel > 60), and sociodemographic variables were determined together with criteria for the diagnosis of S and SO including grip force, lower body strength, appendicular skeletal muscle mass, physical performance, total body fat percentage, body mass index (BMI), waist circumference, and triceps skinfold, together with the levels of salivary cortisol and testosterone. Our results reflected that women presented a higher prevalence of S and SO (21.2% and 30.2%, respectively). A significant difference was observed between salivary testosterone levels and the age of the participants with differences by sex. Testosterone values in men with S and SO were significantly lower (*p* = 0.043 and *p* = 0.048, respectively), which suggests a potential use of the biomarker for diagnostic purposes. No significant differences were shown with cortisol values.

## 1. Introduction

Population aging is a global health problem that mainly affects developed countries. The World Health Organization (WHO) and other international organizations that analyze population health have predicted an increase in the number of older people in the world in the coming decades related to the increase in life expectancy and existing socioeconomic improvements [1]. The aging process is linked to changes in functionality at the cellular, tissue, and physiological levels [2]. Some of the main changes related to this process are the loss of muscle mass and the increase in body fat, often leading to known syndromes and related and more recently diagnosed diseases such as sarcopenia (S) and sarcopenic obesity (SO) [2,3,4]. Thus, according to the European Working Group on Sarcopenia in Older People (EWGSOP), S is defined as a generalized and progressive musculoskeletal disorder associated with a higher probability of falls, fractures, physical disability, and mortality [5]. Regarding SO, the EWGSOP recognizes it as a condition of reduced lean muscle mass in the context of excess adiposity, which mainly affects older people as they are frequently in situations of greater fragility [5]. We know of the existence of biomarkers that could potentially influence the diagnosis and development of sarcopenic disease and SO. However, due to the physiological complexity of these pathologies, it is unlikely that there is a single specific biomarker [6,7] that allows the identification of these diseases in the different vital stages. From this point of view, the study and development of a panel of biomarkers that can harbor direct or indirect relationships in the diagnosis and treatment of said diseases becomes relevant, and these should be easily obtained and interpreted in the clinical context as well as economically viable. For this reason, the use of salivary cortisol (C) and testosterone (T) measurement could be of great help in the early diagnosis of S and SO, since the relationship of C with tissue inflammation and with the breakdown of muscle mass is known [8,9,10,11,12]. In turn, a specific relationship between low T levels with lower muscle mass rates and high inflammation profiles is also known [13,14,15,16].

This study aims to find and evaluate the relationship between altered salivary C and T values and the diagnosis of S or SO in people over 65 years of age. The researchers hypothesized that high C and low T values could be associated with low lean mass, muscle strength deficits, high fat mass, prevalence of sarcopenic disease, and SO.

## 2. Materials and Methods

### 2.1. Study Population

A cross-sectional descriptive study was carried out in over-65s attending municipal activity centers for the older people, which were integrated into the framework of the Chair of Healthy, Active, and Participative Aging signed between the University of Valencia and the City Council of Valencia. In order to avoid considering cases of S secondary to other pathologies and whose inclusion in the study could have interfered with the analysis of the study, all those whose score in the Barthel test [17] would have been less than 60 points were excluded, which ensured that all were independent or slightly dependent on basic activities of daily living (ADLs). The participation was voluntary. None of the older participants were hospitalized during the study, and all resided in their private homes. Participants were informed of this study, which was in accordance with the fundamental principles of the Declaration of Helsinki [18]. This study was approved by the Ethical Committee of University of Valencia (Spain) (number 1139186).

The following inclusion criteria were applied for all participants: (a) be over 65 years of age; (b) be registered at the Senior Citizens Centre; (c) have a Barthel Index equal to or greater than 60 points; and (d) consent to and have the ability to understand and complete all tests included in the study. The exclusion criteria were (a) diagnosis of disease involving severe deterioration of muscle mass to mitigate the effect of secondary S (Parkinson’s disease, Alzheimer’s disease, severe cognitive impairment, stroke, muscular dystrophy, and cancer) and (b) absence at the center on the days of the study.

The initial sample included 322 elderly persons of both sexes, of whom 27 were excluded as being under 65 years of age. Subjects who had a disease involving severe impairment of muscle mass (*n* = 8), those who did not have a Barthel equal to or greater than 60 (*n* = 3), those who were not present on the days of study (*n* = 76), and those who did not complete the study (*n* = 18) were not included in the final sample. The participation rate was 59.01%, and the final sample was 190 people.

### 2.2. Examination Protocol and Measurements

The general information of the participants was collected through an ad hoc questionnaire administered by the researchers. The variables collected were age and sex, the type of cohabitation in the home, and the illnesses suffered by the participants.

### 2.3. Degree of Dependence

The Barthel Index was used only as a secondary screening tool to determine the degree of dependence of the study participants and to avoid possible secondary S cases that could be unnoticed after the exclusion of principal muscular affection diseases.

This index is a validated scale for the Spanish population [17] and of wide clinical use [19,20,21], which considers the subjects that, after the performance of the index, obtain a score of 100 as independent. This study included subjects who scored between 60 (mild dependence degree) and 100 (total independent), since none of these groups presented significant difficulties in the realization of ADLs.

### 2.4. Diagnosis of Sarcopenic Pathology

For the determination of sarcopenic pathology in the study subjects, the latest recommendations of the European Working Group on Sarcopenia in Older People (EWGSOP) [5] of 2019 were used, which integrates three dimensions: low muscle strength [22], low quantity or quality of muscle [23], and low physical performance [24]. These dimensions were analyzed individually with the corresponding tool previously validated. In addition, the SARC-F (sluggishness, assistance in walking, rise from a chair, climb stairs, falls) questionnaire was used to assess the ability to quickly identify S cases [25].

According to the EWGSOP 2019 criteria [5], “probable sarcopenia” was considered for those individuals who only presented low muscle strength (in the lower and/or upper body). For “confirmed sarcopenia”, the above was met along with a low quantity or quality of muscle mass. Finally, if in addition to these two variables, the subjects presented a decrease in physical performance, S was classified as “severe or severe sarcopenia”.

#### 2.4.1. Grip Force (Upper Body Strength)

To determine the force of the upper body, manual ergometry [14,26,27,28] measured with the analogue hydraulic hand dynamometer Jamar 5030J1 was used, with a measuring scale of 0–90 kg/force (kg/f) and an accuracy of 2 kg. The protocol for taking measurements consisted of two attempts for each hand to perform the maximum voluntary grip force, and a one-minute break was taken between each attempt, with the highest score obtained among the four total attempts taken as the final value. The measurements were made with the participants seated in a chair with a straight back and the arm bent at an angle of 90 degrees and being in contact with the trunk. During the measurement, the arm under study was not supported on any surface [29,30]. Values of <27 and <16 kg in men and women, respectively, were considered as indicative of a decrease in force.

#### 2.4.2. Lower Body Strength

To determine the force in the lower body, the test called “sit to stand” with 5 repetitions was used for its practicality and simplicity. This test is included in the test battery proposed by the EWGSOP [5] and consists of making 5 squats into the chair at maximum possible speed and without using any type of manual support [31]. To evaluate the test result, the time used by the subjects in the development of the test was considered. Values greater than 15 s were considered indicative of a decrease in the strength of the participants regardless of sex.

#### 2.4.3. Appendicular Skeletal Muscle Mass (ASMM)

For the determination of ASMM, indispensable for the categorization of S cases, the equation proposed by Kyle et al. [32] was used. In order to develop this formula, we used the results obtained through the electrical bioimpedance carried out with a calibrated digital scale (TANITA DC 430MA-S, Tokyo, Japan; with an accuracy of 0.05 kg) following the latest existing recommendations [33]. Values of <20 and 15 kg in men and women, respectively, were considered indicative of muscle mass decrease (deficit), according to the classification established by the EWGSOP [5].

#### 2.4.4. Physical Performance

The physical performance of the participants was measured by the 4 m speed test [34,35] following recommendations proposed by the EWGSOP [5]. The first test consists of measuring the time it took participants to walk a distance of 4 m at their usual speed, and values of less than 0.8 m/s, regardless of sex, were considered indications of impairment in physical performance. 

In a complementary way, the Short Physical Performance Battery (SPPB) test was also used to determine physical performance. This test consists of performing three tests to assess balance, gait speed, and lower body strength [36,37]. The score and assessment of the total result were the sum of the scores obtained in the three tests. Values lower than or equal to 8 points, regardless of sex, were considered indicative of physical performance deterioration. 

### 2.5. Diagnosis of Obesity

To determine the obesity of the participants, some of the diagnostic criteria that led to the detection of the pathology were used. The selection of these criteria was carried out from an analysis of the latest evidence on SO, detecting great heterogeneity in terms of the methodology related to determination of this pathology [32,38,39,40].

Finally, the diagnostic methods used for the diagnosis of obesity were body mass index (BMI), waist circumference (WC), percentage of total body fat (TBF%), and triceps skinfold (TS).

#### 2.5.1. BMI

To analyze the BMI of the participants, the standard formula (kg/m^2^) was used. Those subjects with a BMI greater than or equal to 30 (kg/m^2^) were considered obese following the WHO criteria [41].

#### 2.5.2. Waist Circumference (WC)

Waist circumference was collected using the criteria of the International Society for the Advancement of Kinanthropometry (ISAK) [42,43] by technicians qualified to perform anthropometric measurements. A retractable inextensible measuring tape was used. Values that were greater than 88 cm in women and 102 cm in men were classified as obesity [41].

#### 2.5.3. Total Body Fat Percentage (TBF%)

Measurement of the percentage of total fat was carried out using bioelectrical impedance analysis (BIA) following the latest existing recommendations to obtain precise measurements [33]. Following Baumgartner’s criterion [44], participants who presented a percentage of total body fat greater than or equal to 38% were considered obese. 

#### 2.5.4. Triceps Skinfold (TS)

The triceps skinfold was evaluated by kinanthropometrists qualified as recommended by the ISAK [42,43], using a Holtain caliper with an accuracy of ±0.2 mm. Values equal to or greater than the 85th percentile according to the criteria presented by Kuczmarski et al. [45] were classified as excess body fat and therefore obesity.

### 2.6. Diagnosis of Sarcopenic Obesity

**Figure 1:**
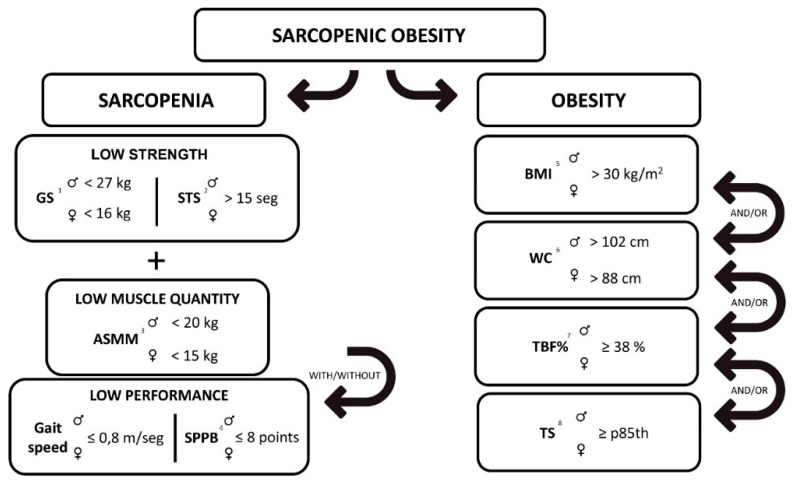
Sarcopenic obesity diagnosis algorithm: ^1^ strength determined by grip strength (GS) and/or ^2^ the sit-to-stand test (STS); muscle quantity determined by ^3^ appendicular skeletal muscle mass (ASMM); low performance determined by gait speed and/or the ^4^ Short Physical Performance Battery (SPPB) test; and obesity determined by ^5^ body mass index (BMI), ^6^ waist circumference (WC), ^7^ total body fat percentage (TBF%), and/or ^8^ triceps skinfold (TS).

In this study, the diagnosis of SO was carried out when obesity and S coexisted in the subjects, according to Oliveira et al. [32], as reflected in Figure 1.

### 2.7. Analysis of Biomarkers in Saliva: Cortisol and Testosterone

The collection of saliva samples for the analysis of the biomarkers was carried out between 10:00 a.m and 12:30 p.m. with the aim of obtaining stable samples that always responded to the same daily interval since many of the hormones found in saliva respond to a circadian pattern that can affect their presence [1,46,47,48,49]. Saliva was collected using a Salivette^®^ kit (SARSTEDT) with a synthetic fiber swab specially developed to detect C and T in saliva. To standardize the sample collection, all the participants were placed in the same time range without having eaten, drank (including caffeinated beverages), brushed their teeth, or practiced physical activity the hour prior to the collection of the salivary sample. Following the manufacturer’s recommendations, the subjects were instructed to place the swab in their mouth without touching it, to keep it in the mouth under the tongue without biting it for approximately 1 min, and return it to the container without handling it. Once collected, the samples were centrifuged for 5 min at 3000 rpm (FUGELAB-GB10 centrifuge) and stored at −20 °C. The centrifuged saliva was analyzed within a month after its collection. T and free C levels were measured using the luminescence immunoassay (IBL, Hamburg, Germany) [50]. 

### 2.8. Statistical Analysis

Statistical analysis was done with IBM SPSS Statistics v.24 software for Windows. To check the normal distribution of data and variables, the Kolmogorov–Smirnov normality test was used; the Student’s T statistic was used when normality was found in the variables, and the U-Mann statistic–Whitney was used when we determined an abnormal distribution. Quantitative variables were presented as means and standard deviations, being analyzed with the Fisher test statistic, while qualitative variables were presented as relative frequencies and percentages.

To search for patterns that established a relationship between C and T levels and the different determining variables in SO and S, bivariate analyses were performed using Spearman’s Rho correlation.

## 3. Results

The total study sample consisted of 190 non-institutionalized older people; 81.5% (*n* = 155) of the participants were women, while the rest, 8.4% (*n* = 35), were men. The mean age of the participants was 72.07 years, with the oldest person being a man of 86 years and the youngest being a woman of 65 years; 61.3% of the women were between 65 and 74 years of age, while 38.7% were older than 75 years. Men 65 and 74 years old comprised 71.4% of the male sample, and 28.6% were older than 75 years. The main characteristics related to the sarcopenic profile of participants are briefly described in Table 1 and Table 2.

Insufficient evidence was found in the literature reviewed on the establishment of reference values for C and T levels in saliva regardless of gender. Consensus was also not found on the units of measurement used in the publications dealing with these biomarkers in saliva; therefore, the authors chose to show both variables as continuous and to use parts per billion (ppb) as the unit of representation of the concentration of these biomarkers. As described in Table 3, the interquartile range for C in women was 0.75 ppb and in men was 0.77 ppb, showing a similar dispersion in data regardless of participants sex. No significant differences were found between both sexes in terms of C levels in saliva (*p* = 0.092).

In the case of testosterone, the interquartile range in women was 0.33 ppb and in men was 0.22, showing less data dispersion in men than in women. If we compare both sexes, significant differences were found between the mean T values (*p* = 0.003). In addition, significant differences were found between the T means in the two age groups analyzed for men, with lower values in the oldest group, a situation that did not occur in the female sex (*p* = 0.038). Comparing the groups according to age range, in the case of C, no significant differences were found in terms of age groups in either sex (*p* = 0.081 in women and *p* = 0.084 in men). Despite this, the mean levels of C in saliva were slightly higher in the younger age groups. Regarding the mean of T levels, women older than 75 years presented higher values despite not finding significant differences (*p* = 0.068). In the group of men, the subjects older than 75 years had a lower mean T than the group of the youngest (*p* < 0.00).

Table 4 shows the mean, interquartile range, and median C and T levels regarding the sarcopenic status of the participants. No significant differences were found between C and sarcopenic state. In contrast, T presented higher and more significant values in the group of men who did not suffer from sarcopenic disease (*p* = 0.043). Regarding SO, its relationship with the biomarkers under study can be consulted in Table 5. 

The subjects with SO showed no significant differences between any of the biomarkers in saliva regardless of sex except for T in the group of men, where the values were lower in the group that presented SO (*p* = 0.048).

Table 6 shows the correlation between the salivary biomarkers studied and the most important variables related to the diagnosis of S and/or SO. A positive relationship was found between C and T regardless of gender, showing higher C levels when higher T levels were found. In the group of women, no other significant relationships were found except for the variable of grip strength, which showed a significant inverse correlation for testosterone; that is, lower T values were found for high grip strength values. In the male group, no significant correlations were found between any of the variables related to the diagnosis of both previously mentioned pathologies and the biomarkers under study.

Table 7 shows a multiple regression (logistic regression) performed to predict changes in the outcomes (S and SO) associated with changes in cortisol and/or testosterone. All associations were non-statistically significant.

## 4. Discussion

Our study found significant differences (*p* < 0.05) regarding the presence of T between the two proposed age groups in the two sexes. These differences were attributed in the case of women to an increase in T associated with age as observed in other existing publications [51,52,53] despite the fact that this relationship has only been found in serum levels. In men, on the contrary, a descending serum pattern associated with age was observed, attributed to a hypogonadism characteristic of the normal aging process, as stated by Saad et al. in their works [54,55] and that coincides with the salivary pattern of this hormone in our publication.

Regarding the presence of C and its relationship with the age and sex of the subjects, no significant differences were found for the proposed groups. In the literature, we found controversy regarding the variation of C as a function of age with works such as Heaney et al. [56], in which no altered salivary C levels were found in older people, and other works such as that of Piazza et al. [57], in which elevated levels of C were found in saliva in older people. We consider that the relationship between C and age is less than the influence that individual pathological processes can exert on variations in the levels of this hormone despite taking into account that the aging process generates a greater amount of low-level inflammation grade in tissues.

The comparison of the mean salivary levels of the biomarkers under study between the groups that presented S or SO differentiated by sex did not present significant differences in terms of the mean amount of C unlike other articles with a relationship in terms of elevated C levels and the incidence of these pathologies (serum values) [6,49,58]. Regarding salivary testosterone, significant differences were found in the group of men, with lower values of this biomarker in the groups affected by sarcopenic pathology or SO, a fact that corresponds to the values obtained by other studies in which serum T (T) and its relationship with low muscle performance and obesity were analyzed [12,13,14,15,54,55,59,60,61,62,63,64]. The nonsignificant results in women could be due to the age-related increase in T and, in turn, to a higher percentage of body fat at menopause [51,52,53,65]. This situation may render the evaluation of this hormone in S and OS invalid for women.

Correlations between the components of S and SO, and salivary C and T levels showed only two significant relationships. The first, a positive correlation between C and T levels, can be interpreted in the case of women as an increase in T associated with the aging process and an increase in C due to metabolic syndrome, as has been observed in some studies [49,51,52,53,65,66]. In the case of men, this situation could not be justified with the existing bibliography, since it shows a decrease in T compared to elevated C levels [67]. Therefore, we attribute these results to possible individual characteristics of the sample.

The second existing significant relationship refers to an inverse correlation between low T levels and greater grip strength in women. An inverse situation to that is found in the available bibliography [54,63,64]. It is important to highlight that, despite this relationship, no significant differences were found between T levels in the group of women with S or SO and the group without pathologies. In this sense, a topic under discussion that has not been analyzed in our study could be the greater physical activity carried out on a regular basis among older women in relation to men, something that has been demonstrated among the Spanish population of older people [68] and that could suppose a possible relationship with the maintenance of strength despite the decrease in T levels, such that lower hormonal levels could come to suppose data related to healthier aging.

As far as we know, this is the first article in which a comparison is made between the results obtained for C and T in saliva and the prevalence of S and SO.

The methodology used to obtain salivary samples could condition the final results of the study, with a potential limitation being the fact that both C and T present differentiated values throughout the day, being highly dependent on the moment in which the measurement is made [16,46,47,48]; this difficulty is solved since the circadian rhythms of these 2 biomarkers are similar and well known, since both steroid hormones show a peak or higher levels during the first hours of the morning and then progressively decrease throughout the day. However, it should not be forgotten that, unlike testosterone, C levels also rise during the first hours of sleep [49] and that these circadian differences can pose a challenge in terms of obtaining representative results for the patient in a diagnosis of any disease that seeks to assess these biomarkers, especially considering the heterogeneity of the sleep-wake patterns of the population [16,46,48,49].

Different publications were found with heterogeneous procedures regarding the collection of saliva samples from C and T [49,66,69,70,71,72,73]. In some articles, the procedure for collecting two or three salivary samples was used to compare the levels of the hormones under study according to the time band [69,70,71,72,73]. In the case of this publication, the researchers chose to use the methodology observed in other articles [49,66,70,71,72] based on the collection of a single salivary sample 3–4 h after waking the participants, thus avoiding the diurnal peak associated with the circadian rhythm of these hormones.

Changes in normal serum C and T values are directly related to the deterioration of muscle mass, S, and SO, as can be consulted in different publications [1,54,55,58,73,74]. The relevant role that these hormones play in terms of muscle mass deficit, cellular stress, and protein degradation makes them a diagnostic target with great potential and need for study through simple and minimally invasive diagnostic methods. The research of these biomarkers in saliva can constitute an easy application test with potential inclusion in the algorithm for the detection and diagnosis of S and SO, which can sometimes be hampered by the deficit of specific biological markers linked to these diseases.

Our work was carried out from a pioneering point of view regarding the search for patterns that would allow the use of salivary biomarkers known for their influence on muscle mass and fat tissue in the diagnosis of pathologies such as S and SO. The algorithm presented by the EWGSOP for the diagnosis of S and the consensus for the diagnosis of SO were followed in detail.

Despite the promising results presented by salivary T as a biomarker taken into account in the diagnosis of S and SO in men, a clear standardization of the protocols for obtaining normalized samples of salivary biomarkers is necessary, especially those with circadian oscillation. An improvement in the sample size of this type of study is also necessary to obtain more representative results.

## 5. Conclusions

Salivary biomarkers can play an important role in the diagnosis and prevention of S. The relationship between T levels in saliva and sex as well as the relationship between this hormone at the salivary level and age in men make it an effective, inexpensive, and easy-to-use parameter, unlike salivary C, which did not yield favorable results. Salivary T is reduced in men with S and SO, which makes it possible to use this biomarker for the adjunctive diagnosis of S and SO. Given that the correlations between the biomarkers under study and the main determinants of sarcopenic pathology and SO did not yield illuminating results from the clinical point of view, it is necessary to broaden this line of research to provide new data that allow for a discussion of the results obtained in this study.

## Figures and Tables

**Table 1:** Sarcopenic status presented by sex and age group according to European Working Group on Sarcopenia in Older People 2 (EWGSOP2) criteria.

		65–75 Years	75–85 Years	Total
Men (*n*)		25	10	35
	Without sarcopenia ^1^	20 (80%)	7 (70%)	27 (77.1%)
	Total sarcopenia ^2^	5 (20%)	3 (30%)	8 (22.9%)
	Sarcopenia probable ^3^	4 (16%)	2 (20%)	6 (17.1%)
	Sarcopenia confirmed ^4^	0 (0%)	0 (0%)	0 (0%)
	Sarcopenia severe ^5^	1 (4%)	1 (10%)	2 (5.7%)
Women (*n*)		95	60	155
	Without sarcopenia ^1^	78 (82.1%)	38 (63.3%)	116 (74.8%)
	Total sarcopenia ^2^	17 (17.9%)	22 (36.7%)	39 (25.2%)
	Sarcopenia probable ^3^	11 (11.6%)	12 (20%)	23 (14.8%)
	Sarcopenia confirmed ^4^	4 (4.2%)	5 (8.3%)	9 (5.8%)
	Sarcopenia severe ^5^	2 (2.1%)	5 (8.3%)	7 (4.5%)
Total (*n*)		120	70	190
	Without sarcopenia ^1^	98 (81.7%)	45 (64.3%)	143 (75.3%)
	Total sarcopenia ^2^	22 (18.3%)	25 (35.7%)	47 (24.7%)
	Sarcopenia probable ^3^	15 (12.5%)	14 (20.0%)	29 (15.3%)
	Sarcopenia confirmed ^4^	4 (3.3%)	5 (7.1%)	9 (4.7%)
	Sarcopenia severe ^5^	3 (2.5%)	6 (8.6%)	9 (4.7%)

^1^ Muscle mass preserved without any degree of muscle dysfunction. ^2^ All cases with any degree of sarcopenic status. ^3^ Reduced muscle strength with preserved muscle quantity or quality and preserved physical performance. ^4^ Reduced muscle strength with low muscle quantity or quality and preserved physical performance. ^5^ Reduced muscle strength with low muscle quantity or quality and low physical performance.

**Table 2:** Mean values ^1^ of the different tests to assess sarcopenia subdivided by sex, age, and sarcopenic status.

		GS ^2^ (kg)	STS ^3^ (s)	ASMM Total ^4^ (kg)	Gait Speed ^5^	SPPB ^6^ (Score)
Men						
	65–75 years					
	Without Sarcopenia	36.5 ± 7.5	10.2 ± 2.4	23.2 ± 3.1	1.1 ± 0.2	10.3 ± 1.5
	Sarcopenia Probable	32.8 ± 5.0	16.2 ± 4.6	25.0 ± 1.3	0.9 ± 0.2	9.0 ± 2.0
	Sarcopenia Confirmed	-	-	-	-	-
	Sarcopenia severe	25.0 ± 0.0	21.0 ± 0.0	16.9 ± 0.0	0.8 ± 0.0	5.0 ± 0.0
	Total Sarcopenia	28.9 ± 5.5	18.6 ± 3.4	20.9 ± 5.7	0.8 ± 0.1	7.0 ± 2.8
	75–85 years					
	Without Sarcopenia	32.7 ± 4.5	12.6 ± 2.7	23.0 ± 2.5	0.9 ± 0.1	9.3 ± 2.2
	Sarcopenia Probable	28.7 ± 6.1	18.9 ± 1.4	24.5 ± 2.4	0.9 ± 0.3	5.3 ± 1.2
	Sarcopenia Confirmed	-	-	-	-	-
	Sarcopenia severe	28.0 ± 1.4	16.5 ± 0.0	19.6 ± 0.3	0.8 ± 0.1	6.5 ± 0.7
	Total Sarcopenia	28.3 ± 0.5	17.7 ± 1.7	22.0 ± 3.5	0.8 ± 0.1	5.9 ± 0.8
Women						
	65–75 years					
	Without Sarcopenia	22.0 ± 3.8	10.3 ± 2.1	16.8 ± 2.4	1.1 ± 0.2	10.4 ± 1.4
	Sarcopenia Probable	16.5 ± 5.8	14.1 ± 4.3	17.9 ± 2.0	1.0 ± 0.3	8.7 ± 1.7
	Sarcopenia Confirmed	12.7 ± 3.2	11.6 ± 1.1	14.2 ± 2.2	1.0 ± 0.1	9.7 ± 2.1
	Sarcopenia severe	15.0 ± 0.0	19.3 ± 0.0	14.4 ± 0.0	0.7 ± 0.0	5.0 ± 0.0
	Total Sarcopenia	14.7 ± 1.9	15.0 ± 3.9	15.5 ± 2.1	0.9 ± 0.2	7.5 ± 2.1
	75–85 years					
	Without Sarcopenia	20.2 ± 3.6	11.0 ± 2.0	15.9 ± 2.2	1.0 ± 0.2	10 ± 1.4
	Sarcopenia Probable	14.9 ± 3.0	17.2 ± 4.8	17.7 ± 2.4	0.7 ± 0.2	7.4 ± 1.7
	Sarcopenia Confirmed	14.8 ± 1.5	12.7 ± 1.7	13.0 ± 2.4	0.9 ± 0.1	10.3 ± 1.5
	Sarcopenia severe	12.6 ± 1.3	15.3 ± 4.6	13.9 ± 1.7	0.8 ± 0.2	7.8 ± 1.1
	Total Sarcopenia	14.1 ± 1.3	15.1 ± 2.3	14.9 ± 2.5	0.8 ± 0.1	8.5 ± 1.6
Men						
	WS ^7^ (all sample size)	34.6 ± 6	11.4 ± 2.6	23.1 ± 2.8	1.0 ± 0.2	9.8 ± 1.9
	TS ^8^ (all sample size)	28.6 ±0.4	18.2 ± 0.6	21.5 ± 0.8	0.9 ± 0.0	6.5 ± 0.8
Women						
	WS ^7^ (all sample size)	21.1 ± 3.4	10.7 ± 2.1	16.4 ± 2.3	1.1 ± 0.2	10.2 ± 1.4
	TS ^8^ (all sample size)	14.4 ± 0.4	15.0 ± 0.0	15.2 ± 0.4	0.9 ± 0.1	8.0 ± 0.7

^1^ Values are presented as mean ± standard error. ^2^ Grip strength. ^3^ Sit to stand test. ^4^ Appendicular skeletal muscle mass. ^5^ Gait speed. ^6^ Short Physical Performance Battery. ^7^ Without sarcopenia. ^8^ Total sarcopenia.

**Table 3:** Descriptive values for salivary cortisol and testosterone by different sex and age groups.

		Women (*n* = 155)				Men (*n* = 35)				*p*-Value *
		65–74 yr	≥75 yr	*p*-Value *	Total	65–74 yr	≥75 yr	*p*-Value *	Total	
		(*n* = 95)	(*n* = 60)			(*n* = 25)	(*n* = 10)			
Cortisol	Mean ± SD (ppb)	1.96 ± 0.77	1.93 ± 0.69	0.081	1.95 ± 0.72	1.98 ± 0.81	1.93 ± 0.68	0.084	1.97 ± 0.76	0.092
	Median (ppb)	1.72	1.62		1.68	1.77	1.82		1.80	
	P25 (ppb) **	1.45	1.34		1.41	1.52	1.55		1.52	
	P75 (ppb) **	2.10	2.22		2.16	2.21	2.66		2.29	
Testosterone	Mean ± SD (ppb)	0.59 ± 0.22	0.66 ± 0.31	0.068	0.63 ± 0.26	0.54 ± 0.19	0.44 ± 0.12	0.038	0.52 ± 0.17	0.003
	Median (ppb)	0.56	0.53		0.56	0.45	0.41		0.45	
	P25 (ppb) **	0.40	0.36		0.38	0.37	0.25		0.34	
	P75 (ppb) **	0.64	0.83		0.71	0.59	0.55		0.56	

* *p*-value < 0.05 was considered statistically significant. ** Interquartile range (percentile 25; percentile 75).

**Table 4:** Descriptive values for salivary cortisol and testosterone by sarcopenic status.

		Women (*n* = 155)			Men (*n* = 35)		
		Sarcopenic (*n* = 39)	Non-Sarcopenic (*n* = 116)	*p*-Value *	Sarcopenic (*n* = 8)	Non-Sarcopenic (*n* = 27)	*p*-Value *
Cortisol	Mean ± SD (ppb)	1.94 ± 0.64	1.95 ± 1.17	0.944	1.72 ± 0.56	2.04 ± 0.77	0.276
	Median (ppb)	1.77	1.63		1.54	1.80	
	P25 (ppb) **	1.49	1.37		1.47	1.56	
	P75 (ppb) **	2.24	2.16		1.83	2.54	
Testosterone	Mean ± SD (ppb)	0.64 ± 0.37	0.63 ± 0.35	0.826	0.44 ± 0.08	0.62 ± 0.32	0.043
	Median (ppb)	0.57	0.55		0.45	0.46	
	P25 (ppb) **	0.41	0.38		0.40	0.31	
	P75 (ppb) **	0.73	0.70		0.50	0.65	

* *p*-value < 0.05 was considered statistically significant and adjusted by sex, age, and BMI. ** Interquartile range (percentile 25; percentile 75).

**Table 5:** Descriptive values for salivary cortisol and testosterone by sarcopenic obesity status.

		Women (*n* = 155)			Men (*n* = 35)		
		Sarcopenic Obesity (*n* = 25)	Non-Sarcopenic Obesity (*n* = 130)	*p*-Value *	Sarcopenic Obesity (*n* = 7)	Non-Sarcopenic Obesity (*n* = 28)	*p*-Value *
Cortisol	Mean ± SD (ppb)	2.03 ± 0.66	1.93 ± 1.12	0.685	1.77 ± 0.59	2.09 ± 0.81	0.293
	Median (ppb)	1.77	1.65		1.61	2.30	
	P25 (ppb)**	1.56	1.39		1.46	1.58	
	P75 (ppb) **	2.24	2.16		1.72	2.54	
Testosterone	Mean ± (ppb)	0.64 ± 0.43	0.63 ± 0.34	0.894	0.53±	0.72±	0.048
	Median (ppb)	0.51	0.56		0.47	0.49	
	P25 (ppb) **	0.41	0.38		0.40	0.31	
	P75 (ppb) **	0.71	0.71		0.52	0.55	

* *p*-value < 0.05 was considered statistically significant and adjusted by sex, age, and BMI. ** Interquartile range (percentile 25; percentile 75).

**Table 6:** Relationship between sarcopenia and sarcopenic obesity variables and salivary biomarkers.

		Cortisol (r_s_ *)	*p*-Value **	Testosterone (r_s_ *)	*p*-Value **
Women	Cortisol	1.000	-	0.363	0.000
	Grip strength	−0.104	0.196	−0.175	0.029
	Sit to stand	0.030	0.713	0.043	0.598
	ASMM	0.040	0.620	−0.039	0.629
	Gait speed	−0.061	0.450	−0.149	0.064
	% Fat mass	−0.006	0.938	−0.088	0.275
Men	Cortisol	1.000		0.354	0.037
	Grip strength	0.114	0.513	0.182	0.298
	Sit to stand	−0.102	0.561	−0.064	0.714
	ASMM	−0.146	0.404	0.047	0.791
	Gait speed	−0.016	0.928	0.204	0.240
	% Fat mass	0.045	0.796	−0.286	0.096

* r_s_: Spearman Rho Correlation. ** *p*-value < 0.05 was considered statistically significant and adjusted by sex, age, and BMI.

**Table 7:** Logistic regression for the prediction of changes in the probability of sarcopenia and sarcopenic obesity associated with changes in cortisol and/or testosterone.

		Sarcopenia OR (95% CI) *			Sarcopenic Obesity OR (95% CI) *	
	Women	Men	Total	Women	Men	Total
Cortisol	0.54	0.97	0.93	0.54	1.08	1.01
	(0.13–2.34)	(0.67–1.41)	(0.64–1.34)	(0.13–2.31)	(0.73–1.60)	(0.68–1.51)
Testosterone	0.31	1.15	1.03	0.31	1.01	0.87
	(0.01–18.3)	(0.40–3.32)	(0.37–2.87)	(0.005–18.3)	(0.29–3.56)	(0.61–3.76)

* OR (95% CI): odds ratio per change in 1 ppb (95% confidence interval).

## Data Availability

The data presented in this study are available on request from the corresponding author.
